# Towards the Interpretation of Sound Measurements from Smartphones Collected with Mobile Crowdsensing in the Healthcare Domain: An Experiment with Android Devices

**DOI:** 10.3390/s22010170

**Published:** 2021-12-28

**Authors:** Robin Kraft, Manfred Reichert, Rüdiger Pryss

**Affiliations:** 1 Institute of Databases and Information Systems, Ulm University, 89081 Ulm, Germany; manfred.reichert@uni-ulm.de; 2 Department of Clinical Psychology and Psychotherapy, Ulm University, 89081 Ulm, Germany; 3 Institute of Clinical Epidemiology and Biometry, University of Würzburg, 97078 Würzburg, Germany; ruediger.pryss@uni-wuerzburg.de

**Keywords:** mHealth, crowdsensing, tinnitus, noise measurement, environmental sound

## Abstract

The ubiquity of mobile devices fosters the combined use of ecological momentary assessments (EMA) and mobile crowdsensing (MCS) in the field of healthcare. This combination not only allows researchers to collect ecologically valid data, but also to use smartphone sensors to capture the context in which these data are collected. The TrackYourTinnitus (TYT) platform uses EMA to track users’ individual subjective tinnitus perception and MCS to capture an objective environmental sound level while the EMA questionnaire is filled in. However, the sound level data cannot be used directly among the different smartphones used by TYT users, since uncalibrated raw values are stored. This work describes an approach towards making these values comparable. In the described setting, the evaluation of sensor measurements from different smartphone users becomes increasingly prevalent. Therefore, the shown approach can be also considered as a more general solution as it not only shows how it helped to interpret TYT sound level data, but may also stimulate other researchers, especially those who need to interpret sensor data in a similar setting. Altogether, the approach will show that measuring sound levels with mobile devices is possible in healthcare scenarios, but there are many challenges to ensuring that the measured values are interpretable.

## 1. Introduction

Smart mobile devices (e.g., smartphones) are becoming increasingly ubiquitous. Their capabilities allow the combined use of ecological momentary assessments (EMA) and mobile crowdsensing (MCS) in the healthcare domain to not only collect qualitative longitudinal and ecologically valid data, but also to use sensors of smartphones as well as connected external sensors (e.g., wearables) to capture the context in which these data are collected [1]. For example, environmental data (e.g., noise [2,3]) can be measured when a questionnaire is answered to correlate the questionnaire data with the environmental data to gain new insights about patients. However, sensor measurements must be accurate, comparable, and interpretable to provide meaningful information. Especially for non-standardized smartphone sensors like the microphone (i.e., different manufacturers, different mobile operating systems, different scales), it can be challenging to achieve these properties.

The TrackYourTinnitus (TYT) mobile platform uses EMA and MCS to track a user’s individual tinnitus. Tinnitus is the perception of an internal sound in the ears in the absence of a corresponding external sound. As symptoms are subjective and vary over time, TYT was created to monitor and evaluate the variability of these symptoms in the daily life of tinnitus affected patients or interested users [4]. The platform has been in operation since 2014 and is composed of a registration and information website (https://www.trackyourtinnitus.org/, accessed on 1 October 2021), a central backend for data storage, and a mobile application available for both Android and iOS. The mobile apps assess users’ individual tinnitus perceptions (e.g., tinnitus loudness and distress) by asking them to complete tinnitus EMA questionnaires at different times of the day [5]. In addition, the environmental sound level is captured in parallel with the completion of the daily questionnaire [5]. The detailed process of the TYT app is described in [1], whereas the underlying data set (i.e., structure and insights to the collected data) is described in [6]. The overall objective of this work is to investigate the correlations between environmental sound level and reported tinnitus symptoms. More specifically, it should be examined whether the environmental sound level has an effect on tinnitus. If the sound levels can be correlated to questionnaire-collected data, new insights might be unveiled as the sound level data can be considered more objective than data from completed questionnaires alone (e.g., to allow predictions on tinnitus loudness based on the sound data). In this context, further note that, for tinnitus and many other diseases and disorders, longitudinal studies that are able to collect ecologically valid data for such a long time are still very rare. In addition, the collection of objective data succh as the sound level is even more scarce. Since TYT has been running for more than half a decade, and not all circumstances of the collection procedure were clear to the developers beforehand, it is now of great interest to make the collected amount of sound levels interpretable from a medical perspective. Therefore, the experiment at hand is important for TYT, but the results and lessons learned may be of much greater value for the healthcare domain in general.

However, the data available in the TYT database [6] do not contain calibrated sound pressure level (SPL) or weighted decibel (e.g., dB(A)) values, but rather relative amplitude (Android) or uncalibrated decibel (iOS) values as retrieved from the mobile system APIs. This fact prevents a direct comparison of these values and therefore a meaningful interpretation regarding the correlation with tinnitus symptoms. A preceding calibration of the mobile devices and storing respective dB_SPL_, dB(A) or dB(C) values would circumvent this issue. To encounter that sound sensor values measured by a smartphone require further considerations in healthcare scenarios is also recognized by other works than TYT [7]. From a general viewpoint, sensor measurements collected by a modern smartphone for healthcare purposes require many considerations before collected sensor data can be actually evaluated. In [8], for example, challenges are discussed in the context of fall detection. One of the challenges discussed by the authors of [8] also has implications for the data collected by TYT, namely the usability when collecting sensor data. If a user has his or her smartphone in the pocket, collected sensor values may not be usable. Consequently, works can be found that try to mitigate such challenges on a more generic level [9]. However, the data in the TYT database were collected for more than six years with more than 100,000 entries, and the respective mobile apps used to collect these values cannot be changed retroactively to counteract the described issues. Since no other works could be found that helped to analyze these pre-existing collected sound pressure levels, the following requirements were established for the experiment shown in the work at hand:

- Identification of an experimental setting that can be used to learn more about the interpretation possibilities of the collected TYT sound level values.
- In addition to the latter point, in the best case, the experiment should be appropriate to enable us to compare all sound level values across the different smartphone devices from different manufacturers and different mobile operating systems.
- Conduction of the experiment without the use of an expensive sound laboratory, with the goal to foster and facilitate the overall reproducibility.

Based on these requirements, different scenarios have been discussed. In the end, the following approach (i.e., list of decisions for the experiment) was conceived to make the described values usable and comparable:

1. The TYT database was analyzed to identify the mobile device models that contributed the most environmental sound measurement data.
2. The analysis of the database showed that more detailed device information is available for Android devices. For this reason, it was decided to use Android devices for the experiment.
3. A sample of the identified device models was selected and acquired (i.e., we purchased these devices for the experiment).
4. A new mobile application was developed that mimics the behavior of the TYT app with respect to the sound measurement. More specifically, the app was implemented with the specific focus on the sound measurement but using the same software functions as TYT (i.e., by copying the relevant source code fragments from the original app).
5. The selected device models were equipped with this mobile application.
6. For the evaluation of the smartphone devices equipped with the app, a sound signal was generated, for which the volume was adjusted to different sound levels using a professional calibrated sound level meter (SLM). Based on this setting, the values captured by the mobile app on the different mobile devices were recorded.
7. Finally, the results were used to derive equations for the different device models that, in turn, can be used to transform the measurement data in the database into (partially) comparable dB(C) values.

How these steps were carried out in practice and what results were achieved are discussed in the following sections. In Section 2, a detailed discussion of related works will be presented. Section 3 presents the experiment in detail, while Section 4 presents its results. A discussion of the results with respect to limitations and practical relevance will be provided in Section 5. Section 6 closes our work with a summary and an outlook for future work.

## 2. Related Work

Measuring sound levels with smartphones has been a topic of research for some time. There are both scientific and commercial implementations of apps that perform sound measurements. In addition, studies evaluating the accuracy and precision of these apps can be found in the literature. Moreover, the ability of smartphones to perform sound level measurements in the environment as well as their calibration has been investigated and discussed in a thorough manner. Finally, there are works that deal with large data sets of sound levels measured with smartphones.

NoiseMap [10] is an Android app that performs geo-referenced sound measurements and sends these data to an open urban sensing platform following a participatory sensing approach to create real-time noise maps and data graphs. The incoming sound signal is sampled and first translated to a relative dB full scale (dBFS) value and subsequently to a dB_SPL_ value by adding a constant calibration value. A built-in calibration tool can be used to determine this value using a constant pink noise [10]. The iOS app SoundLog [11] was developed by the Australian National Acoustic Laboratories (NAL) with the aim to provide a personal noise dosimeter. The app is capable of measuring A-weighted equivalent continuous sound levels (LA_eq_), C-weighted peak sound pressure levels (LC_pk_), as well as other values for different sampling periods [11]. Ambiciti [12] is a mobile app developed for both Android and iOS that utilizes mobile crowdsensing to enable urban noise monitoring. The app performs automatic background noise measurements in dB(A) using the microphone and the user’s location. In addition, a calibration feature is provided [12]. The accuracy of the app has been evaluated and found to be within ±1.2 dB(A) [13]. The City Soundscape [14] mobile app is used as part of a noise monitoring platform in the context of acoustic urban planning in smart cities. The app mimics the user interface of a professional SLM and is able to measure dB_SPL_ and equivalent continuous sound level (L_eq_) values [14]. Furthermore, there are numerous apps implementing sound measurements available in the Google Play Store (e.g., refs. [15,16,17]) and the Apple App Store (e.g., refs. [18,19,20]). However, in the context of environmental and occupational noise monitoring, for most of these apps there is no information available on the algorithms used as well as no systematic and standardized evaluation of their quality and accuracy, which is a common issue in the field of mHealth apps [21]. There are various studies evaluating the accuracy of existing apps [22,23,24,25,26,27,28,29]. These studies were thereby either conducted in controlled laboratory environments [22,23,24,25,27,28,29] and used pink noise [23,24,28,29], white noise [25,27,28,29], 1/3 octave band noise [22], or representative audio samples [29] to simulate sound sources with different sound levels, or were performed in real-world field environments [26,28]. Results indicate that some sound measurement smartphone apps may be considered accurate and reliable to a certain degree (±1 dB(A) or ±2 dB(A) respectively), but most of the apps cannot be used as reliable tool to assess the environmental sound [23,25]. In general, iOS apps performed better than Android apps, which can be attributed to the fact that Android devices are built by several different manufacturers and there is a lack of conformity of microphones and other audio components [23,25]. It has been shown that accuracy can be improved if the smartphone apps are calibrated before the measurements [27]. Furthermore, it has been shown that the use of an external calibrated microphone can further increase the accuracy and precision of sound measurements compared to measurements using internal smartphone microphones [30].

Moreover, the ability of smartphones to perform environmental sound level measurements in general has been extensively discussed in the literature [31,32,33,34]. In [32], the sound capture and processing procedure when using smartphones for environmental noise measurements is investigated by analyzing the impact and accuracy of different algorithms, time periods, and sampling strategies for noise calculation. The results indicate that, with the correct settings, it is possible to measure noise levels in the range of 35–95 dB(A), with an accuracy of ±2 dB(A). Other studies have shown that an adequate sound level meter smartphone app that is used together with an external microphone can achieve compliance with most of the requirements of Class 2 of the IEC 61672/ANSI S1.4-2014 standard for periodic testing [33], as well as full compliance for directional response in the horizontal plane [34]. The authors of [31] discuss the use of smartphones in the context of urban noise pollution and present a field-study evaluating the relevancy and accuracy in this context. The results indicate that smartphones can be used as useful noise measurement devices with an accuracy of ±3 dB(A) if careful review of the collected data is undertaken.

Furthermore, the calibration of smartphones for sound measurements and different approaches in this regard have been discussed in this context [35,36,37,38,39]. In [35], a laboratory calibration method for noise measurement smartphone apps is presented based on frequency response linearization and an A-weighted sound level correction. The authors of [36] introduce a calibration method that does not require user interaction and is based on a node-based calibration utilizing a linear model and a common indoor quiet noise base. Slow-start issues of this approach are mitigated with the help of a crowdsourcing-based calibration. A cross-calibration method for participatory sensor networks based on outlier detection, crowd sensors-based correction, fixed sensors-based correction, and day–evening–night noise level (L_den_) estimation is proposed by [37]. In [38], an averaging method for the calibration of a smartphone microphone against a reference microphone in terms of sound pressure level and frequency spectrum measurements is presented. It is shown that the method can be used to calibrate a smartphone using another smartphone calibrated using the same method. Finally, the authors of [39] propose a calibration method for smartphones that does not require specific equipment or knowledge of the user by utilizing the low variability of the average noise emission of vehicles.

Finally, works that deal with large data sets of sound levels measured with smartphones can be found in the literature. For example, interpolation [40,41] and simulation [41] strategies for producing sound maps based on such smartphone measurements have been investigated and discussed in this context.

However, to the best of our knowledge, the evaluation of an pre-existing large data set of uncalibrated environmental sound level amplitude values measured with smartphone sensors has not yet been considered in the literature. In this context, the chosen approach of making the data set of sound measurements comparable and interpretable by taking a sample of devices from this data set, calibrating them, and deriving corresponding equations is a novelty. Furthermore, none of the existing related works considers the assessment of environmental sounds measured with smartphone sensors, or smartphone sensor measurements in general, in the context of tinnitus.

## 3. Materials and Methods

First, the materials and methods used to perform the experiments in the scope of the work at hand are described. In this context, the data set used for the initial analysis is outlined. Furthermore, the selection of hardware and software components used for the experiments is described. Finally, the experimental setup and procedure are delineated.

### 3.1. Data Set for the Analysis

The data set for the analysis has been extracted from the TYT database on 26 January 2020 and contains a total of 76,542 entries. The structure of the TYT data set has been described in [6]. In this data set, 45,712 (59.72%) entries belong to an Android device, 30,607 belong to an iOS device (39.99%), and 223 of the entries contain no user agent information (0.29%), as shown in Table 1. As described in [6], for every answer sheet that is collected with the TYT mobile applications for Android and iOS, the user agent is extracted and stored together with the answer data. For the Android version of the app, this user agent contains, among other information, the constant Build.MODEL from the android.os.Build API (https://developer.android.com/reference/android/os/Build#MODEL, accessed on 1 October 2021), which can be used to uniquely identify the respective device model (see Table 2). Note that for the iOS version of TYT, only the device type (iPhone/iPad) and the OS version is stored in this variable. For this reason, it was decided to use Android devices for the experiments in the scope of this work.

Furthermore, a sound level measurement capturing the environmental noise level for the first 15 s of the user completing the EMA questionnaire is performed and stored together with the EMA answer data. For the Android version of the app, this value represents an amplitude value retrieved by the Android MediaRecorder API [42] and averaged over the measurement period. The Android source code that was used in the application to retrieve this value is later analyzed and discussed in Section 4.2. In contrast, the iOS version stores a relative dB value, which is not further analyzed in the scope of this work.

### 3.2. Hardware and Software Selection

The selection of the hardware as well as software used for the experiments is described in the following. This includes the selection process used to decide on the mobile devices to be investigated. In addition, other relevant hardware and software used to perform the experiments themselves, namely the sound level meter, calibrator, speaker, tone generator, and the mobile application for the sound measurement, are described.

#### 3.2.1. Mobile Devices

In order to perform the experiments for an optimal subset of devices that allows assumptions to be made about as many entries in the data set as possible, the data set described in the previous section was analyzed from two different perspectives.

For the first analysis, the data set was analyzed on a *per-device* basis. To this end, the following procedure was used:

1. For each entry, the device IDs of the device models (see Section 3.1) are extracted.
2. For each extracted device ID, the number of unique users and entries containing a sound measurement are counted.
3. For each device ID, the device names are looked up and device IDs with the same device name are summarized in a new row.

The 30 most used device models resulting from this process are shown in Table 2.

For the second analysis, the data set was analyzed on a *per-user* basis with regard to the intended interpretation of the data. Thereby, users (and their respective device models used) were selected based on the following conditions:

- There are more than 500 entries containing sound measurements for the user.
- The reported tinnitus loudness (see [6]) is fluctuating and appears plausible (e.g., not only zero values and not always the same value).
- The sound measurement is fluctuating and appears plausible (e.g., not only zero values and not always the same value).

Finally, the identified devices from both analyses were combined, resulting in eight devices, as highlighted in Table 2. Since the selected device models had to be purchased and not all devices were available at the time of starting the experiments, only four of the eight identified devices could be used (highlighted in dark gray in Table 2). On top of these four devices, a *Google Pixel 2* was used simply because it was available to the experimenters. This resulted in the five devices shown in Table 3. The Android version installed on each device can be found in the table. These are the maximum versions that were officially supported by the acquired devices at the time of the experiments.

#### 3.2.2. Reference Sound Level Meter and Calibrator

As a reference sound level meter (SLM) for the performed sound measurements the *testo 815* by *Testo SE & Co. KGaA* is used. It allows measurements in the range of 32 to 130 dB and a frequency range of 31.5 to 8000 Hz. The SLM supports frequency weightings A and C. Its accuracy is ±0.5 dB under reference conditions at 94 dB and 1000 Hz in accordance with Class 2 of IEC 60,942 [43], with a resolution of 0.1 dB. In order to avoid distortions due to differences in temperature and air pressure, the sound level calibrator PeakTech 8010 by *PeakTech Prüf- und Messtechnik GmbH* was used to calibrate the SLM. The accuracy of the calibrator is ±0.5 dB under reference conditions at 23 ${}^{\circ }$C, 1013 mbar air pressure and 65% humidity.

#### 3.2.3. Speaker and Tone Generator

As a sound source for the experiments, the speaker of the *GigaWorks T20 Series II* by *Creative* connected to a notebook was used. The Online Tone Generator by Tomasz P. Szynalski [44] was used on the notebook to generate a sine wave (pure tone) on different frequencies.

#### 3.2.4. Mobile Application for Sound Measurement

**Figure 1:**
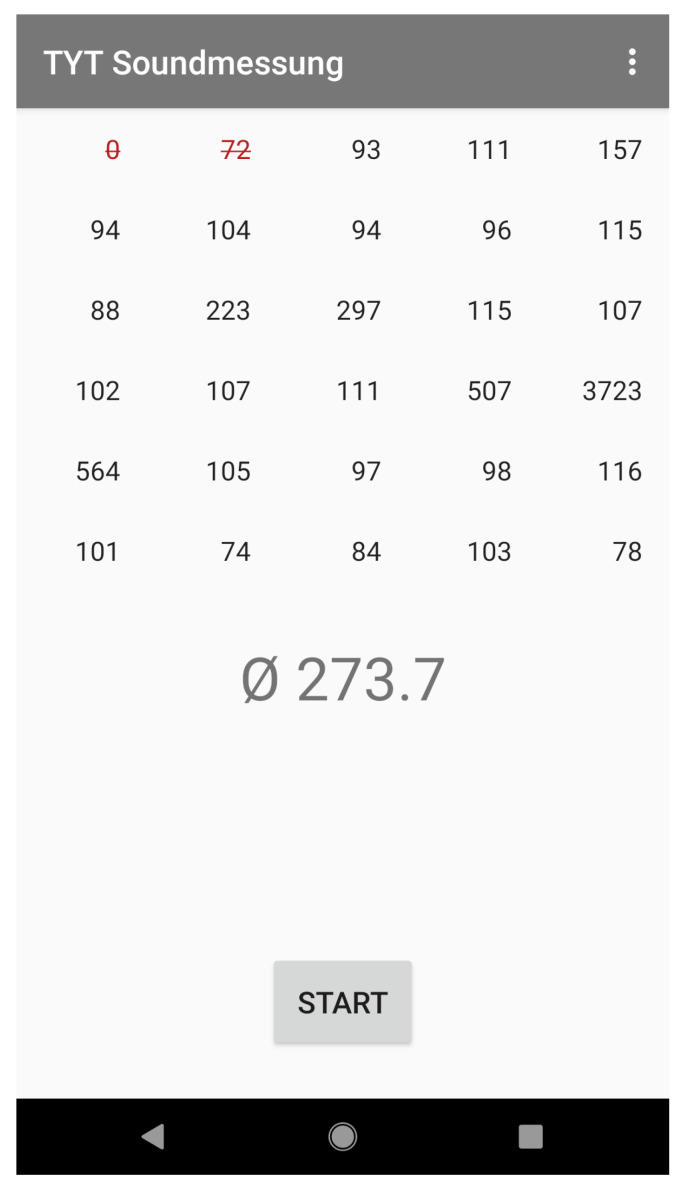
Screenshot of the sound measurement mobile application used for the experiments. The values displayed represent the individual amplitude values for each of the 500 ms periods as well as the average amplitude over the entire 15 s measurement period (the large number in the center of the screen).

In order to mimic the behavior of the TYT app for the experiments, the corresponding code for the sound measurement was extracted and integrated into a new sound measurement mobile application. In addition, this allows to implement a more convenient way of extracting the results, as well as more insights into various parameters of the sound measurement. Equivalent to the TYT app, the sound measurement application utilizes the previously described MediaRecorder.getMaxAmplitude() method to capture the “maximum absolute amplitude that was sampled since the last call to this method” [42] every 500 ms for a total of 30 values (15 s). These values, in turn, are then averaged into a single value. This averaging step was found to be erroneous in the original application, as will be discussed in Section 4.2, and has been corrected for the application used in the experiments. Furthermore, the first two values of the sound measurement have shown to be erroneous for several smartphone models (see Section 4.2) and are therefore discarded for the measurements. A screenshot of the sound measurement application is shown in Figure 1. The user interface of the application allows to start the measurement and displays the measured single amplitude values as well as the resulting average value after the measurement is done. As shown in the screenshot, the first two values that are discarded and excluded from the average are highlighted by displaying them as crossed out in red. In addition to the features used for the experiments in the scope of this work, the application allows further configurations for experimental purposes (e.g., the option to change the audio encoding as well as to remove any audio compression) and offers the possibility to perform a continuous measurement of the sound level.

### 3.3. Experimental Setup and Procedure

Before conducting the actual experiments, various measurements were taken with different frequencies (125–2000 Hz), frequency weightings (A & C), distances to the sound source, and different smartphones to find the optimal settings for the experiments. The measurements indicate that—using the correct settings—the smartphones measure sound frequency-independently in the study’s frequency range of 125–2000 Hz, allowing a single frequency to be used for the experiments. The final settings are shown in Table 4. A pure tone with a frequency of 1000 Hz was chosen for the sound source to obtain an unweighted result with the given SLM, since it supports only A- and C-weightings and these frequency weightings do not apply offsets at 1000 Hz [45]. Note that, for this reason, dB_SPL_, dB(A) and dB(C) at 1000 Hz are all equal and may therefore be used interchangeably for measurements at this frequency. For purposes of clarity, dB(C) is used for the remainder of this paper. To promote and facilitate the overall reproducibility, it was decided against a professional sound laboratory in favor of a simpler test environment for the experiments. Thus, for the measurement range, a lower limit of 50 dB(C) was chosen because the background noise in the test environment was measured at approximately 46 dB(C). 80 dB(C) was chosen as upper limit to avoid hearing damage for the experimenter (without additional protective measures). A distance of 30 cm between sound source and SLM/smartphone was chosen due to spatial restrictions to avoid reflections in the test room.

**Figure 2:**
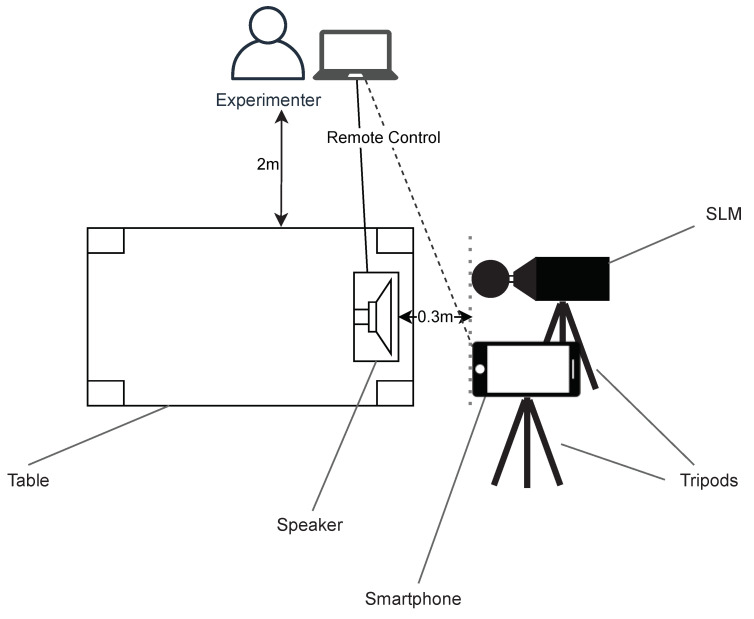
Setup for the experiments.

The experimental setup is shown in Figure 2. The experiment is performed in a room of 15 square meters. The speaker is positioned at the edge of a 76 cm high table to avoid reflections by the table surface. Furthermore, it is fixated in a way that accounts for its slightly upward design and results in a vertical positioning of the speaker cone. The SLM and each of the smartphones are screwed onto tripods and positioned as close as possible to each other and 30 cm from the speaker, with their microphones pointed at the speaker. The SLM is thereby rotated 90 degrees so that its display can be read from a distance by the experimenter. The speaker and the smartphone are controlled remotely with a notebook that is positioned 2 m away from the table to avoid reflections by the equipment and the experimenter.

Before conducting the experiments, the SLM is calibrated with the calibrator to account for the room conditions such as temperature and air pressure. Thereby, the calibrator is attached to the SLM and turned on, producing a sound at 94 dB and 1000 Hz. The SLM is then configured to measuring range 50–100 dB, time weighting “Fast” (the measured samples are averaged every 125 ms) and frequency weighting A. The SLM is then potentially fine-tuned until the display also shows 94 dB.

The experimental procedure is structured as follows and was repeated for each of the five smartphones.

1. The tone generator software is used to create a 1000 Hz sinus signal (pure tone) with the speaker.
2. The volume is then adjusted until the SLM shows the desired sound pressure level.
3. Subsequently, the measurement is started on the smartphone. As described in Section 3.2.4, the mobile application captures 30 measurement values (while discarding the first two values) for about 15 seconds, averages these values and stores them in a table.
4. The steps 1.–3. are repeated for 5 dB increments between 50 and 80 dB(C) (an explanation for the measuring range can be found in the first paragraph of this subsection), resulting in seven values per smartphone.

## 4. Results

**Figure 3:**
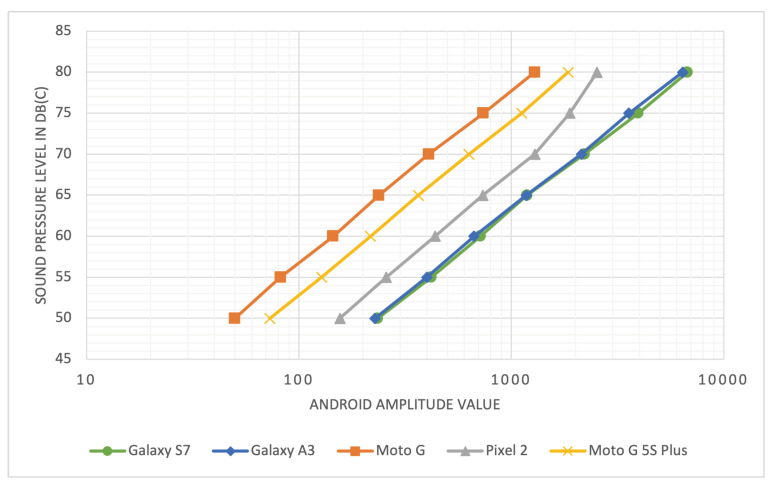
Measured amplitude values for C-weighted sound pressure levels between 50–80 dB(C) for the different mobile devices used in the experiments. The x-axis is logarithmically scaled.

The final experiments resulted in a total of 35 values. The results are shown in Figure 3. The y-axis shows the reference dB(C) value produced with the tone generator and the speaker. On the x-axis, the output of the different smartphone models is displayed on a logarithmic scale. It can be seen that the measured amplitudes of all smartphone models show an almost linear slope on the logarithmic scaled axis, indicating a nearly logarithmic slope of the values. Furthermore, it can be observed that the curves of the smartphones are almost parallel, indicating that the slopes are nearly identical. The only noticeable deviation is shown by the Pixel 2, where the curve seems to bend at 70 dB(C). Overall, the curves appear to differ only by an offset on the x-axis.

The results of the experiments are then analyzed in terms of their interpretation. In this context, first, the experimental results are used for a logarithmic regression to derive respective equations for the different device models. Second, the legacy application code of the TYT app is analyzed for relevant implementation errors and poor design decisions that should be improved. Finally, the derived equations are used to transform the existing data in the TYT database into (partially) comparable dB(C) values.

### 4.1. Deriving Equations from the Experimental Results

**Figure 4:**
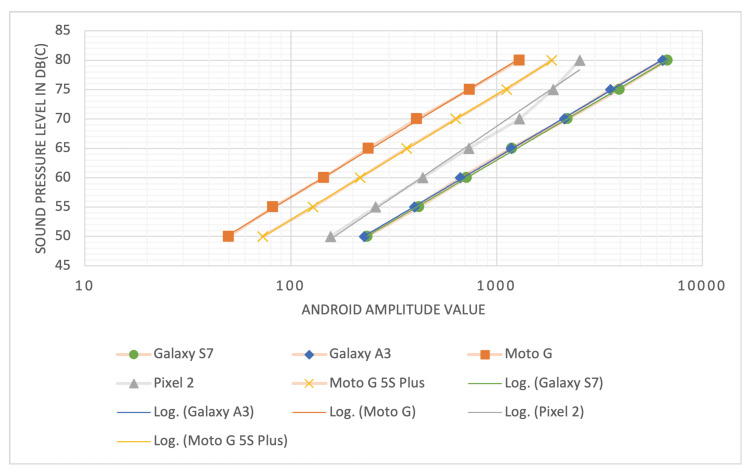
Fitted regression curves for the measured sound levels of the different device models. The x-axis is logarithmically scaled, resulting in linear curves.

As can be seen in Figure 3, the curves for each device model have approximately the same slope. A logarithmic regression analysis was performed to fit a logarithmic function to the relationship between amplitude values of each device model and the respective sound level in dB(C) measured with the SLM. The resulting equations are listed in Table 5 and plotted on top of the measured data in Figure 4. As can be seen in the table and the figure, the regression curves have similar slopes (s=1.33), but differ in their intercept. Only the Samsung Galaxy S7 and A3 models seem to have an almost identical curve, which suggests that the manufacturer used the same or similar hardware and software components for the devices. For the other device models, the results indicate that the Android devices process sound levels equally except for an offset of 0–15 dB. Furthermore, the slopes of the equations appear to be similar to that of the definition of sound pressure level (SPL), shown in Equation (1), where *p* is the root mean square sound pressure and $p_{0}=20\;\mu$ Pa = 2 $\cdot 10^{-5}\;$ Pa is the reference sound pressure [46]. For sound measurements by the device models used in the experiments, the equations from Table 5 can be used to transform an amplitude value of the respective device model into a corresponding dB(C) value. As the slopes of the equations are similar, a simple calibration of any additional device model in order to determine the respective offset might already be sufficient in order to obtain approximately comparable measurements.

(1) $$L_{p}=10\cdot \log_{10}\left(\frac{p^{2}}{{p_{0}}^{2}}\right)dB=20\cdot \log_{10}\left(\frac{p}{p_{0}}\right)dB$$

### 4.2. Analysis of the Legacy Application Code

As mentioned in Section 3.2.4, the code of the TYT mobile application that is used to measure the sound level values that are later stored in the database was analyzed and tested in an isolated environment before the beginning of the experiments. Thereby, several errors were found in the process used to obtain these values, which are briefly described in the following:

- Erroneous calculation 1: The 30 amplitude values sampled by the app as retrieved by the Android MediaRecorder API are averaged arithmetically and stored as a single value, which is supposed to represent the average sound level. This is erroneous, as sound levels are logarithmic values, which must be transformed to their energetic source values before they can be used for calculations [45].
- Erroneous calculation 2: The first two measured values of the app often contained errors in the initial experiments. For multiple of the investigated devices, the first measured value was consistently 0, while the second value was often too low. Further experiments showed that these errors occur very frequently for measurements within the first 1000 ms after the start of the recording. These findings indicate that these first two values should be excluded from the calculations.
- Unsuitable audio codec: The audio encoder AMR_NB [47] is used for the measurements, which is a narrowband audio codec optimized for a frequency range between 200 and 3400 Hz [48]. Lower and higher values may therefore be recorded in a distorted manner.
- Lack of user transparency: The app does not indicate that the sound measurement is ongoing. The user could therefore interact with the mobile device in an unfavorable way, which might interfere with the measurement (e.g., microphone is covered, smartphone collides with object). For example, interacting with the touchscreen of the mobile device during the measurement resulted in an increase of the measured sound level by about 10 to 20 dB(C). Placing the device on a table led to values above 100 dB(C).

To estimate the magnitude of the error due to the erroneous calculation, a worst case was simulated, for which 29 of the 30 measured amplitude values are used as input for the calculation that are rather small and one value that is rather high. We chose the amplitude values measured for 50 dB(C) and 80 dB(C) respectively, as these were the lowest and highest sound level values used in the experiments. The resulting dB value that would be calculated by the TYT app as well as the correct dB value are shown in Table 6. These values can be interpreted to mean that the sound level values stored in the TYT database are up to 9.4–9.8 dB lower than the actual measured loudness. Note that a difference of 10 dB is perceived as approximately double loudness [45]. Therefore, the measured values in the TYT database cannot be considered as representative environmental noise measurement in dB_SPL_ (or dB(C), respectively), and thus cannot be used for corresponding conclusions. However, the values could still be used to compare them relatively (e.g., lower and higher sound levels) and to investigate correlations with other data (e.g., the perceived tinnitus loudness of a single user).

### 4.3. Interpretation of the Existing Data

The equations from Section 4.1 can be used to transform the soundlevel data from the TrackYourTinnitus database (see Section 3.1) for the respective device models into (partially) comparable sound level dB(C) values (although these values are erroneous, as shown in Section 4.2). Table 7 shows the minimum (min), maximum (max) and average (avg) dB(C) values for the amplitude values stored for the device models. Note that noise exposure of 85 dB(A) over a period of 8 hours is considered hazardous [49].

## 5. Discussion

In the following, the results are discussed. On the one hand, considerations towards comparability of sound measurements with smartphones are discussed. On the other hand, limitations of the experiments in the scope of this work are considered.

### 5.1. Towards Comparability of Sound Measurements with Smartphones

The results have shown that measuring sound levels with mobile devices (e.g., smartphones) is possible if the devices are calibrated correctly beforehand. However, there are several aspects that should be considered. The mobile application used to measure the sound level should be carefully revised regarding the following aspects:

- If system-APIs are used, it should be verified whether these APIs provide the correct values and whether these values are in the desired format. If the recording requires a setup time, the measurement should only be started after this setup is completed.
- Audio codecs that distort the measurements should not be used.
- Consideration should be given to whether average or peak values are of interest.
- If sound level averages are calculated, the logarithmic nature of the amplitude values must be taken into account and the correct formula must be used.
- The mobile application should transparently indicate via the user interface that the sound measurement is in progress to avoid the user unintentionally interacting with the mobile device in a way that interferes with the measurement. The user should be instructed to act appropriately to minimize the interference.

### 5.2. Limitations

The experiments performed in the scope of this work are subject to several limitations. First, the measurements were not performed in a laboratory to foster and facilitate the overall reproducibility. Therefore, measurement errors, especially due to sound reflections or background noises (e.g., traffic noise), might have distorted the results. Second, the measuring distance of 30 cm from the sound source was chosen for spatial reasons. It was not verified whether a greater distance would lead to more accurate measurement results. Third, the measurements were limited to levels between 50 and 80 dB(C). Values below or above these limits cannot be verified. Fourth, the measurements were performed with a single sinus signal (pure tone) sound source at 1000 Hz. Generalizations for other sound signals and different frequencies might not be accurate. In addition, pure tones might lead to room modes and standing waves that could have distorted the results, which was not considered in the experiments. Fifth, in this context, dB(C) values as measured by the SLM are treated as dB_SPL_ in the experiments, which cannot be generalized for frequencies other than 1000 Hz. Furthermore, for environmental noise measurements usually the A-weighting filter is used to better reflect the hearing of the human ear. Sixth, the output of the mobile application is a peak value and not an effective value as measured by the SLM. These values should not be compared directly, but were nevertheless used to simulate the behavior of the TYT app. Seventh, it is assumed that the Android API used to retrieve the amplitude values behaves the same on each Android version, since the experiments were performed with the maximum version that was officially supported by the acquired devices (see Table 3). This assumption is supported by the fact that the API has been present since Android API level 1 (Android 1.0) [42], but could not be verified.

## 6. Summary and Outlook

In this work, an experiment was described with the objective to make a large data set of environmental sound measurements captured with smartphones and stored in the TrackYourTinnitus (TYT) database usable and comparable to enable meaningful interpretations in the context of tinnitus research. To this end, the existing data were analyzed to find the device models that contributed the most data entries. Four of these device models were then acquired for the experiments and equipped with a mobile app that mimics the environmental sound measurement of the TYT Android app. For the actual experiments, a sound signal was generated, the volume was adjusted to different sound levels using a professional calibrated sound level meter (SLM), and the values captured by the source code of the app on the Android devices were recorded. The results indicate that the amplitude values retrieved by the devices behave similarly except for a constant offset. Furthermore, equations derived from the results with a logarithmic regression analysis can be used to transform the values in the TYT database to (partially) comparable dB values. However, there are several limitations to the experiments due to the code of the TYT app and the experimental setup.

Since the experiments within the scope of this work were only conducted for a number of selected Android device models, in future work, more device models should be considered. This includes both Android as well as iOS device models. For the latter, there are far fewer different models, which are all produced by a single manufacturer, which simplifies the process. Once the values retrieved by the system APIs of the different device models and operation system versions are known, respective equations can be derived and used for any future measurements of the same models. Alternatively, along with the recommendations in Section 5.1, a calibration feature could be integrated in a future version of the TYT app that could lead to even more accurate results.

In conclusion, it has been shown that measuring sound levels with mobile devices is possible and feasible for healthcare purposes, but there are many challenges to ensuring that the measured values are accurate, comparable, and interpretable and thus more future work towards the interpretation of mobile crowdsensing data should be conducted.

## Figures and Tables

**Table 1:** Data set from the TYT database used for the analysis.

Description	Entries	%
Total	76,542	100
Android	45,712	59.72
iOS	30,607	39.99
No user agent information	223	0.29

**Table 2:** The 30 most commonly used mobile device models, ordered descending by the number of measurements for each device model in the TYT database. The devices that were selected to perform the experiments are highlighted in gray. The devices in light gray were initially selected but could not be used because we were not able to acquire them.

Device ID	Device Name	Number of Users	Number of	*⌀* Measurements
			Measurements	per User
	Moto G *	9	2113	235
	Galaxy S5 *	38	1779	47
LGL34C	LG Optimus Fuel	1	1548	1548
	Galaxy S3 mini *	15	1491	99
SM-G800F	Galaxy S5 mini	14	1173	84
XT1032	Motorola Moto G	7	1113	159
	Galaxy S3 *	28	1059	38
GT-I8190	Galaxy S3 mini	8	1030	129
SM-G900F	Galaxy S5	24	998	42
GT-I9300	Galaxy S3	23	858	37
	Galaxy S9 *	24	838	35
SM-A300FU	Galaxy A3	2	779	390
HTC One	HTC One	9	776	86
GT-I9195	Galaxy S4 mini	10	756	76
	Galaxy S4 mini *	11	756	69
SM-A520F	Galaxy A5	6	637	106
PRA-LX1	Huawei P8 Lite	1	620	620
WAS-LX1A	Huawei P10 Lite	4	614	154
SM-C115	Galaxy K Zoom	1	606	606
Moto G	Moto G	1	510	510
SM-T810	Galaxy Tab S2	2	506	253
XT1028	Moto G	1	490	490
	Galaxy S4 *	30	464	15
SM-G960F	Galaxy S9	16	462	29
LG-P970	LG P970	1	461	461
LT26i	Sony Xperia S	3	458	153
Moto G (5) Plus	Moto G5 Plus	2	446	223
SGH-M919	SGH-M919	3	444	148
	Galaxy S7 *	21	442	21
	Galaxy S6 *	30	433	14

* marks device models that summarize multiple device IDs under a common device name (e.g., “Moto G *” summarizes the device IDs “Moto G”, “XT1028” and “XT1032”). These models can appear both as a single device model and as part of their group.

**Table 3:** Final selection of device models and respective installed Android versions used for the experiments.

Device Name	Device ID	Android Version
Moto G	XT1032	5.1
Galaxy A3	SM-A310F	7.0
Galaxy S7	SM-G930F	8.1
Moto G 5S Plus	Moto G (5S) Plus	8.1
Pixel 2	Pixel 2	10.0

**Table 4:** Settings for the experiments.

Parameter	Value
Sound source	
Frequency	1000 Hz
Sound pressure level	50–80 dB(C)
Increments	5 dB
Distance	30 cm
SLM	
Frequency weighting	C
Time weighting	Fast
Measuring range	32–80

**Table 5:** Equations for the different device models as results from the logarithmic regression. R^2^ is the coefficient of determination.

Device Model	Logarithmic Regression Model Equation	R^2^
Galaxy S7	$y=20.5379\cdot \log_{10}\left(x\right)+1.3481$	0.9995
Galaxy A3	$y=20.7228\cdot \log_{10}\left(x\right)+1.2215$	0.9997
Moto G	$y=21.216\cdot \log_{10}\left(x\right)+14.258$	0.9994
Moto G 5S Plus	$y=21.341\cdot \log_{10}\left(x\right)+10.166$	0.9998
Pixel 2	$y=23.8364\cdot \log_{10}\left(x\right)-2.7633$	0.9925

**Table 6:** Worst case errors due to the erroneous calculation of the TYT app for the dB(C) values used in the experiments.

Device ID	Device Name	dB(C) Value TYT App	dB(C) Value (Correct)	Difference (Error)
SM-G930F	Galaxy S7	55.9	65.3	9.4
SM-A310F	Galaxy A3	55.9	65.5	9.6
XT1032	Moto G	55.9	65.6	9.7
Moto G (5) Plus	Moto G (5) Plus	55.4	65.2	9.8

**Table 7:** Minimum, maximum, and average recorded environmental noise levels in the TYT database for the different device models used in the experiments.

Device ID	Device Name	dB(C) Min	dB(C) Max	dB(C) Avg
SM-G930F	Galaxy S7	31.0	94.7	79.1
SM-A310F	Galaxy A3	38.4	93.4	81.1
XT1032	Moto G	40.4	105.4	82.7
Moto G (5) Plus	Moto G (5) Plus	52.6	93.9	78.0

## Data Availability

The data presented in this study are available on request from the corresponding author. The data are not publicly available due to privacy reasons.
